# Annotations

**Published:** 1887-12-10

**Authors:** 


### ANNOTATIONS.

**Mrs. Partington at the "Lancet" Office.**

THE Editors of the *Lancet* appear to have delayed their holidays until November this year. They have evidently all been absent from duty at the same time, and have left their journal in the charge of what we may hope is the only Dame Partington in the medical profession. The good dame has taken a riotous advantage of the opportunity, and has shaken her mob-cap and flourished her broom in such a very excited fashion as to amuse and amaze, if not to enlighten, a considerable section of the *Lancet's* readers. We have a certain degree of sympathy with the object our contemporary has in view, which appears to be the warning of all whom it may concern against the dangers of what may be called "self-medical treatment by means of journalism." It has, however, with a reprehensible reticence which the profession and all honest men must resent, failed so far to name the papers it attacks, and so judgment on the merits is impossible. We ourselves, though only partly a medical journal, constantly warn our non-medical readers against the same thing; indeed, on commencing our new series on October 1st of the present year, we went so far as to state in the leader (page 2) that we should "make no attempt to take the line of teaching every man to be his own doctor; the man who is his own doctor has a fool for his patient." But whilst so far agreeing with our venerable, if somewhat reactionary contemporary, we cannot think that the purpose it has in view will be served by what may appear to non-professional readers to be the mere angry vapourings of an interested proprietor, who scents mischief in the air in the shape of a possibly diminishing circulation and a gradual contraction in the bulk of the advertisement sheets. We would venture to point out to our contemporary that it is a little late in the day in taking up the cudgels, and that it has left out of account certain offenders who, more publicly and brazenly even than wicked journalists, try to spread abroad certain principles of "health for the people." We refer, of course, to *Ambulance Lecturers*, who pursue their nefarious occupation in every town; *Health Lecturers*, who are constantly at work in Edinburgh, and many other large and enlightened centres, under the auspices of the medical professsion, and who often are themselves professors and assistant professors of medical schools which have the power of conferring degrees. These "ambulance" and "health" lecturers appear to go even further than those journals which the *Lancet* so detests, for they boldly address public assemblies of hundreds of persons at once, of both sexes and all ages, and tell them plainly, not only what to do in emergencies, but also in all kinds of slight ailments, such as colds, headaches, constipation, chilblains, nervous excitement, journalistic fogeyism, and we know not what. Besides, there are those who affirm that not only is the *Lancet* itself one of the chief offenders in "touting" for a "lay" circulation, but it actually led the way: else why is it found every week publicly exposed for sale at Messrs. Smith and Sons' bookstalls and on the counters of booksellers' shops? It surely cannot be considered light reading, or its illustrations specially appropriate for young lady travellers! The West-end and other clubs may, perhaps, properly be regarded by the proprietors as fitter spheres for certain portions of its contents. At any rate, it would be difficult to find a club or reading-room of any kind where this "purely professional journal" is not "taken." The real object of the *Lancet* in its virtuous leaders—lacquered over as they are with the feeble cant of an affected professional brotherliness—is so transparently obvious that we will not even take the trouble to point it out. We would venture also to remind our contemporary that it not infrequently happens, by the thaumaturgic energies of a new time and new ideas, that a curse is turned into a blessing. In any case it seldom does the individual cursed any harm. Every one remembers how a certain black-coated gentleman—to wit, the Jackdaw of Rheims—lived through all the most solemn terrors of "bell, book, and candle," and remained a flourishing jackdaw nobody knows how long—

> "Never was heard such a terrible curse;
> But what gave rise
> To no little surprise
> Nobody seemed one penny the worse."

For ourselves, we take the opportunity of repeating our warning that "the man who is his own doctor has a fool for his patient." But we will not make ourselves so ridiculously futile as to threaten our offending brethren with the penalties which may be visited upon them by offended University Senates and Examining Boards, because if we did they would merely laugh at us, knowing full well that many members of those very tribunals have themselves committed the terrible sin of giving publicly, without fee, to some general instructions in the principles of health, including the common treatment of common ills. None but babies are frightened by "bogies," and even they will clap their hands merrily when the "bogey" displays all the characteristic features of a wizened and toothless "fogey."

**State-Supported Doctors.**

In the items of the last budget of the kingdom of Saxony is a grant of 52,000 marks towards the support of physicians in the poorer districts of the kingdom. Such an enlightened step as this well worthy of imitation in our own country, in the interest both of doctors and patients. There is a complaint from many districts of Saxony that the people are too poor to support a doctor—a complaint which might be echoed by many districts, both urban and rural, in Britain. There can be no doubt that in this country many people die from lack of proper medical attendance. It is the honest and hard-working poor who are most chary of calling in a medical man. They dread the expense of his visits and of the medicine he may prescribe, and so, when one of the family falls ill, they do their unskilled best for the patient—a best that is often the worst possible treatment—till the disease assumes a serious complexion, and they call in the doctor, often only in time to sign the death certificate. To be sure, there is always the parish doctor; but his services cannot be obtained without the whole family being set down as paupers, a word which conveys a cruel and undeserved stigma; for there is many a household whose members can just keep their heads above water while they are in health, but whose resources cannot bear the expense of a long illness, and who, while they are not paupers, but the most independent and self-respecting of the community, are wholly unable to pay for competent medical attendance, while they are too honest to call in a doctor and leave his bill unpaid. On the doctor, too, these circumstances react unfavourably; for a man who has spent much time and a large sum of money in learning the work of his profession naturally expects to make by it an income that shall be something more than genteel beggary, and so devotes himself to curing the ailments of some wealthier parts of the community. This leaves the poorer districts to be doctored by some less competent physician, whose diploma has been more easily obtained; or, worse, by one who by some action that has forfeited the respect of the profession or the public, has unfitted himself for lucrative and honourable practice. We do not say that all the doctors in our poorer districts belong to one or other of these classes; far from it. East-end London and barren country districts can show physicians who are heroes, and often martyrs to duty; but all cannot be of such a noble strain. Would it not be well, then, to encourage doctors to settle in poor districts by such an amount of State support as would afford them a sufficient living? We admit the need of the State supporting clergymen to minister to the spiritual welfare of the people; surely it would be well to follow the example of the Saxon Government, and accord a similar support to those who care for the body.

